# Sodium channel-inhibiting drugs and cancer survival: protocol for a cohort study using the CPRD primary care database

**DOI:** 10.1136/bmjopen-2016-011661

**Published:** 2016-09-06

**Authors:** Caroline Fairhurst, Fabiola Martin, Ian Watt, Tim Doran, Martin Bland, William J Brackenbury

**Affiliations:** 1 Department of Health Sciences, University of York, York, UK; 2 Hull York Medical School, York, UK; 3 Department of Biology, University of York, York, UK

**Keywords:** Anticonvulsants, Breast neoplasms, Colonic neoplasms, Prostatic neoplasms, Sodium channels

## Abstract

**Introduction:**

Voltage-gated sodium channel (VGSC)-inhibiting drugs are commonly used to treat epilepsy and cardiac arrhythmia. VGSCs are also widely expressed in various cancers, including those of the breast, bowel and prostate. A number of VGSC-inhibiting drugs have been shown to inhibit cancer cell proliferation, invasion, tumour growth and metastasis in preclinical models, suggesting that VGSCs may be novel molecular targets for cancer treatment. Surprisingly, we previously found that prior exposure to VGSC-inhibiting drugs may be associated with reduced overall survival in patients with cancer, but we were unable to control for the cause of death or indication for prescription. The purpose of the present study is to interrogate a different database to further investigate the relationship between VGSC-inhibiting drugs and cancer-specific survival.

**Methods and analysis:**

A cohort study using primary care data from the Clinical Practice Research Datalink database will include patients with diagnosis of breast, bowel and prostate cancer (13 000). The primary outcome will be cancer-specific survival from the date of cancer diagnosis. Cox proportional hazards regression will be used to compare survival of patients taking VGSC-inhibiting drugs (including antiepileptic drugs and class I antiarrhythmic agents) with patients with cancer not taking these drugs, adjusting for cancer type, age and sex. Drug exposure will be treated as a time-varying covariate to account for potential immortal time bias. Various sensitivity and secondary analyses will be performed.

**Ethics and dissemination:**

The project has been reviewed and approved by the University of York Ethical Review Process. Results will be presented at an international conference and published in open access peer-reviewed journals according to the STROBE and RECORD guidelines.

**Strengths and limitations of this study**

- The data source is a large prospectively collected primary care database containing information on causes of death, comorbidities and drug exposure based on prescription data.
- Potential immortal time bias will be considered and a person-time approach implemented.
- Cancer stage and other confounding details of the cancer and secondary care treatments are not available.
- Data on severity of epilepsy are not available.
- General practitioner records may be of variable quality and invariably contain incomplete data.

## Introduction

Voltage-gated sodium channels (VGSCs) are composed of large pore-forming α subunits (Na_v_1.1–Na_v_1.9) and smaller auxiliary β subunits (β1–β4).^1^ VGSCs regulate action potential firing, growth and migration of electrically excitable cells, including neurons and myocytes.^2–5^ Abnormal VGSC function is frequently a contributing factor in a number of excitability-related disorders, including epilepsy, cardiac arrhythmias, depression and neuropathic pain.^6^ ^7^ Thus, a number of commonly prescribed antiepileptic drugs and class Ib antiarrhythmic agents, including phenytoin, lamotrigine, carbamazepine and valproate, elicit their therapeutic effect by inhibiting the conductance of VGSCs.^8^

VGSCs are widely expressed in a number of major cancers, including carcinomas of the breast, prostate and colon.^9–11^ A number of studies have shown that VGSC activity promotes the migration and invasion of metastatic cancer cells in vitro.^12–23^ Silencing Na_v_1.5 expression in breast cancer cells with shRNA inhibits tumour growth and metastasis in an orthotopic xenograft breast cancer model in mice.^24^ On the other hand, overexpression of the β1 subunit increases metastasis.^25^ ^26^ Furthermore, the VGSC-inhibiting antiepileptic drug phenytoin significantly reduces migration, invasion and metastasis of cancer cells in preclinical models, suggesting that VGSCs may be novel molecular targets for treating metastasis.^27–29^

We recently conducted a systematic review of clinical and preclinical studies testing the effects of VGSC-inhibiting drugs in cancer.^30^ Only two clinical studies were identified that explored the effect of VGSC-inhibiting drugs in patients with cancer, and their effect on survival and metastatic relapse was not clear.^31^ ^32^ However, we identified 22 preclinical studies collectively, suggesting that a number of VGSC-inhibiting drugs inhibit various aspects of cancer progression, including proliferation, angiogenesis and invasion, suggesting that further investigation in this area is urgently required.^27^ ^28^ ^33–52^

Using data from the QResearch primary care database, we recently considered the hypothesis that exposure to VGSC-inhibiting drugs which started prior to cancer diagnosis may lead to an increased time to metastasis and thus improved survival time in patients with breast, bowel and prostate cancer.^53^ We selected all patients from the database with a diagnosis of breast, bowel or prostate cancer and a prescription for a VGSC-inhibiting drug prior to their cancer diagnosis. This group formed the exposed group. The unexposed group consisted of a random sample of patients with a breast, bowel or prostate cancer diagnosis who had never had a recorded prescription for a VGSC-inhibiting drug. Data on the cause of death were not available and so our primary outcome was all-cause mortality. In contrast to our hypothesis, and the preclinical evidence, we found that patients with cancer prescribed VGSC-inhibiting drugs prior to their diagnosis had an increased all-cause mortality rate relative to the control group.^54^ However, we could not rule out the possibility that the elevated mortality of the exposed group was due to confounding by indication, for example, epilepsy diagnosis, or differences in baseline frailty.

The purpose of the present study is to use a different data set from the Clinical Practice Research Datalink (CPRD), in which we have access to data on causes of death and life-limiting comorbidity diagnoses, in order to test the hypothesis that exposure to VGSC-inhibiting drugs may predict altered survival of patients with cancer. The objectives are to investigate:

- the relationship between VGSC-inhibiting drug use and cancer-specific and overall survival, controlling for potential confounding factors, for example, epilepsy and baseline frailty,
- the relationship between timing of VGSC-inhibiting drug exposure (eg, before vs after cancer diagnosis) and overall and cancer-specific survival.

## Methods and analysis

### Data source and sample selection

We will perform a retrospective cohort study using individual patient data accessed from the CPRD database (https://www.cprd.com/home/), a governmental, not-for-profit research service holding anonymised primary care records drawn from participating general (primary care) practices dating back to 1987, which can be used for public health research. Records were sampled in two stages. First, 125 general practices were purposively selected to be nationally representative in terms of area deprivation and list size. Second, up to 2500 patients were randomly sampled from each practice. The final data set contains records for ∼289 000 patients. These data have been specifically selected to be a large, general and nationally representative data set for use by multiple research groups collaborating with members of the Department of Health Sciences, University of York, to test a range of hypotheses. We will use these data to investigate the mortality of patients with breast, bowel or prostate cancer stratified by whether or not they have a recorded prescription of a VGSC-inhibiting drug. Data relating to patient characteristics, general practice, medical codes, clinical/consultation details, referrals, immunisations, tests and therapies (prescriptions and treatments) are available. We will identify and extract the records for all patients with a diagnosis of breast, bowel or prostate cancer (hereafter referred to as the index cancers) received before 31 December 2014 to allow at least 1 year of follow-up for all patients. In practice we will define a cancer diagnosis as any recording of one of these cancers as a Medical Code (medcode, CPRD unique code for the medical term selected by the general practitioner (GP), linked to Read codes) in the clinical data set of GP consultations in the CPRD (code list available at clinicalcodes.org). The date of first mention of the cancer in the records will be used as a proxy for the date of diagnosis, since the cancer is likely to have been diagnosed previously in a secondary care setting. The CPRD primary care data that we will use are not linked to cancer registry data, such as staging and secondary care therapy (eg, chemotherapy); therefore, these data will not be available for this study. Patients aged younger than 25 at the time of diagnosis will be excluded as it is unlikely a person of that age would have one of these three cancers. Causes of death are available and include an underlying cause of death and, often several, contributory causes.

### Exposure

We will identify which patients with cancer have (ever had) a recorded prescription for a VGSC-inhibiting drug (table 1). For each prescription, the product name, substance, strength, formulation (tablet, gel, injection etc), route of administration, British National Formulary (BNF) code and header and date of administration are recorded. The BNF header contains the licensed indication(s) for the substance, and so does not necessarily indicate the purpose for which the prescription was administered to the specific patient at that specific time. The definition of exposure to VGSC-inhibiting medication will initially include prescriptions for lidocaine injections, which are often used as a local anaesthetic. These will be excluded from the primary analyses since we believe that these injections will have only a transient effect and are not likely to have the same effect as longer term prescriptions for other VGSC-inhibiting drugs.^53^ However, we will compare the outcomes of patients whose VGSC-inhibiting drug prescriptions are only for lidocaine injections following their cancer diagnosis with the unexposed group because there is evidence that local anaesthetics used perioperatively associate with reduced tumour recurrence.^55^

**Table 1:** VGSC-inhibiting drugs

Drug	Alternative names	Target/mechanism	BNF section
Amiodarone	Cordarone X	VGSC inhibitor	2.3.2 Antiarrhythmic drugs
Carbamazepine, eslicarbazepine, oxcarbazepine	Arbil, Carbagen SR, Epimaz, Inovelon, Tegretol, Teril, Timonil, Trileptal, Zebinix	VGSC inhibitor	4.8 Antiepileptic drugs
Disopyramide	Dirythmin, Isomide, Rythmodan	VGSC inhibitor	2.3.2 Antiarrhythmic drugs
Dronedarone	Multaq	VGSC inhibitor	2.3.2 Antiarrhythmic drugs
Flecainide	Tambocor	VGSC inhibitor	2.3.2 Antiarrhythmic drugs
Lacosamide	Vimpat	VGSC inhibitor	4.8 Antiepileptic drugs
Lamotrigine	Lamictal	VGSC inhibitor	4.8 Antiepileptic drugs
Lidocaine	Lignocaine, Xylocard	VGSC inhibitor	2.3.2 Antiarrhythmic drugs
Mexiletine	Mexitil	VGSC inhibitor	2.3.2 Antiarrhythmic drugs
Moracizine	Ethmozine	VGSC inhibitor	–
Phenytoin, fosphenytoin	Epanutin, Pentran	VGSC inhibitor	4.8 Antiepileptic drugs
Procainamide	Pronestyl	VGSC inhibitor	–
Propafenone	Arythmol	VGSC inhibitor	2.3.2 Antiarrhythmic drugs
Quinidine	Kiditard	VGSC inhibitor	–
Ranolazine	Ranexa	VGSC inhibitor	2.6.3 Other antianginal drugs
Riluzole	Rilutek	VGSC inhibitor	4.9.3 Drugs used in essential tremor, chorea, tics, and related disorders
Rufinamide	Inovelon	VGSC inhibitor	4.8 Antiepileptic drugs
Tocainide	Tonocard	VGSC inhibitor	–
Topiramate	Topamax	VGSC inhibitor	4.8 Antiepileptic drugs
Valproate, valproic acid	Convulex, Depakote, Epilim, Epival, Episenta, Orlept	VGSC inhibitor	4.8 Antiepileptic drugs
Zonisamide	Zonegran	VGSC inhibitor	4.8 Antiepileptic drugs

The most commonly prescribed class of drug for each patient will be identified. The extent of drug exposure will be estimated by calculating the time between the first and last recorded prescription, plus a number of weeks to account for the time they were assumed to be taking the drug from their final recorded prescription (calculated as the average observed interval between prescriptions for all exposed patients).

### Outcome measures

Metastasis is the cause of 90% of deaths from solid tumours;^56^ however, metastasis is not reliably recorded in primary care data. Therefore, we shall investigate time to metastasis, where possible, as a secondary outcome but our primary outcome measure will be cancer-specific survival (where *any* cancer is listed as the *underlying* cause of death) following cancer diagnosis. Other secondary outcome measures will include (1) *any* cancer listed among *any* of the causes of death; (2) *index site-specific* cancer listed as the *underlying* cause of death; (3) *index site-specific* cancer listed among *any* of the causes of death and (4) all-cause mortality.

- A cancer death will be identified as having a derived underlying cause code of C00-D09 in ICD-10 or 140-209 in ICD-9.
- A prostate cancer death will be identified as having the derived underlying cause code of C61 in ICD-10 or 185 in ICD-9.
- A breast cancer death will be identified as having the derived underlying cause of C50 in ICD-10 or 174 or 175 in ICD-9.
- A bowel cancer death (which includes cancers of the colon, rectum and rectosigmoid junction) will be identified as having a derived underlying cause of C18-C20 in ICD-10 or 153 or 154 in ICD-9.

### Confounding factors

We will consider the following confounding factors:

- Other life-limiting disease indications for VGSC-inhibiting medication listed in the BNF: epilepsy, cardiac arrhythmias, amyotrophic lateral sclerosis and neuropathic pain/painful neuropathy. Patients with a recorded mention of one of these medical codes will be identified using Read codes;
- Comorbidities: the Charlson Comorbidity Index will be calculated for each patient using Read codes for each condition;^57^ ^58^
- Ethnicity will be identified using Read codes, and from linked CPRD and HES data, and will be categorised as white, mixed, Asian or Asian British, black or black British, other and unknown;
- Body mass index will be identified using Read codes. Patients will be categorised as having low (<20), normal (20–24.9) or high (25+) BMI, or will be placed into an ‘unknown’ category where no data are provided. The patient's BMI recorded at the closest date to the date of index cancer diagnosis will be used in the analysis;
- Level of physical activity will be identified using Read codes. Patients will be categorised as undertaking limited/light exercise, or regular exercise, according to their status as recorded at the closest date to the date of index cancer diagnosis;
- Alcohol intake will be identified using Read codes. Patients will be categorised as non-drinker, ex-drinker, light drinker (<3 units/day), moderate drinker (3–6 units a day), heavy drinker (7+ units a day) or unknown. Where alcohol use is implied but no level of consumption is recorded, then the patient will be assumed to be a moderate drinker. The alcohol status recorded at the closest date to the date of index cancer diagnosis will be used in the analysis;
- Smoking status will be identified using Read codes. Patients will be categorised as non-smoker, ex-smoker, light smoker (<9 cigarettes a day), moderate smoker (10–19 cigarettes a day), heavy smoker (20+ cigarettes a day) or unknown. Where it is implied that the patient is a smoker but no level of consumption is recorded, then the patient will be assumed to be a moderate smoker. The smoking status recorded at the closest date to the date of index cancer diagnosis will be used in the analysis.

### Sample size calculation

Since we shall be working with a fixed data set, the size of the sample will ultimately depend on the number of patients in the data set with a recorded diagnosis of breast, bowel or prostate cancer. Sample size determination for survival studies is a complex procedure. We present here a basic calculation using parameter estimates obtained from our previous study and based on formulae presented by Shoenfeld.^59^ We have previously estimated a multivariable-adjusted HR for death from any cause among VGSC-inhibiting drug users, compared to patients in the unexposed group, to be 1.42 (95% CI 1.36 to 1.49, p<0.001), indicating a statistically significant increased risk of death in the exposed group.^54^ To detect this relative hazard with 80% power, given a conservative ratio of 20 unexposed patients per exposed patients and a significance level of 5%, requires 1344 events. Given a baseline event rate of 0.04 for the unexposed group, an average follow-up time of 5 years and a censor rate of 0.3 requires 686 patients in the exposed group and 13 003 patients in the unexposed group. Shoenfeld advises that this calculation may not be reliable for non-randomised studies where covariates are likely to be extremely imbalanced between the treatment groups; however, covariates were observed to be similar between the exposed and unexposed groups in our previous study.^54^

### Statistical analysis

Analyses will be conducted in Stata (StataCorp LP, College Station, Texas, USA, version 13), using two-sided significance tests at the 5% level. Participants with anomalous, incorrect or infeasible dates will be excluded, for example, dates of cancer diagnoses recorded before birth or after death. Cox proportional hazards regression models to analyse survival time from cancer diagnosis will be adjusted for drug exposure, type of cancer, gender and age at diagnosis (age will be included as a linear and quadratic term (age+age^2^)) unless otherwise stated. Multivariable-adjusted HRs will be presented with a 95% CI and p value.

Cox regression assumes that the proportional hazards model applies. To assess this, we shall plot −log(−log(S(t))) against log(time), where S(t) is the survivor function at time t. The curves for the two groups should be parallel. We will also consider a χ^2^ test of the Schoenfeld residuals to assess the null hypothesis of no relationship between the hazards in each group. If the assumptions are not met, we shall try to investigate why this is.

### Primary data set

Participants in the CPRD data set with a recorded medical code of one of the three index cancers diagnosed before 31 December 2014, and aged over 25 years at diagnosis.

### Descriptive summaries

The characteristics of the comparison groups will be described using summary statistics. Categorical data will be presented as frequency and percentage, and continuous variables will be summarised using descriptive statistics (mean, SD, median, minimum and maximum). Comparisons between the groups will be made using χ^2^ tests and t-tests (or Wilcoxon rank-sum tests) as appropriate. A flow diagram will present the selection and exclusion (with reasons where possible) of patients in the study. Length of exposure and the most commonly prescribed class of drug for each patient will be summarised. The underlying causes of death will be categorised as cancer, ischaemic heart disease (I20–I25 in ICD-10 and 410 to 414 in ICD-9) and other, and summarised for the two groups. The number of deaths where epilepsy is stated as a cause will also be reported; however, a limitation is that many deaths related to epilepsy will be recorded as a different cause, such as accident or suicide, and it will not be possible to definitively attribute epilepsy to these deaths.

### Replication of previous analysis

We shall replicate our initial analysis of data from the QResearch database as closely as we can.^53^ ^54^ This will involve identifying patients in the primary data set who were aged 30 years or older at registration. From this cohort, patients with at least one recorded prescription for a VGSC-inhibiting drug prior to the earliest diagnosis of an index cancer will be identified, and prescriptions for lidocaine removed—this group will make up the exposed group. All patients in the primary data set, who were aged 30 years or older at registration and who do not have a recorded prescription, will make up the unexposed group. The distribution of time from cancer diagnosis to death (all cause) will be described using Kaplan-Meier survival estimates for the two groups. The statistical equivalence of the two curves will be examined using the log-rank test. Survival from cancer diagnosis will be compared between the exposed and unexposed groups using an adjusted Cox proportional hazards regression model. We will also stratify the analysis to consider survival in the following two groups of patients: (1) those whose prescriptions ended before their cancer diagnosis and (2) those whose prescriptions continued after their diagnosis.

### Primary analysis

**Figure 1:**
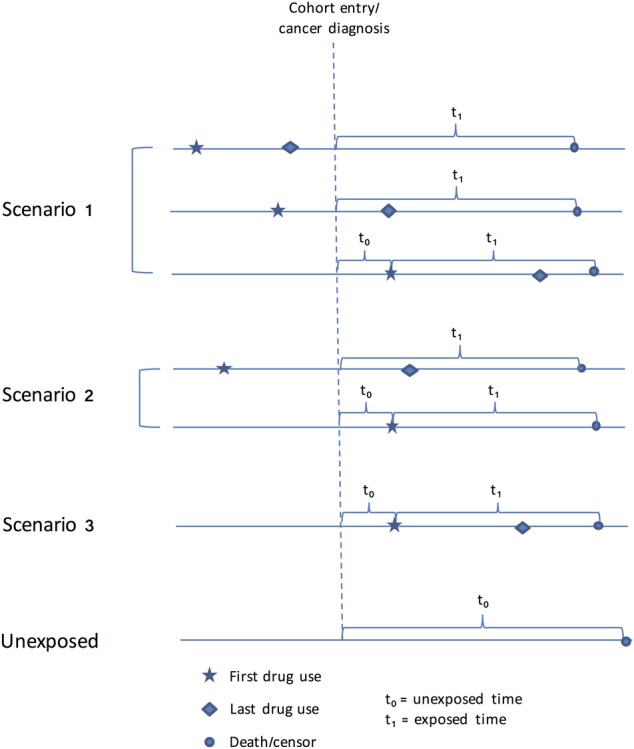
Description of typical exposed and unexposed participants in this cohort study, and the three ways we will define drug exposure in the time-dependent analyses. Scenario 1: person-time will be considered as unexposed until the date of first prescription (whether before or after diagnosis) and as exposed thereafter. Scenario 2: person-time will be considered as unexposed until the date of first prescription and as exposed thereafter for patients whose prescriptions either: (1) start before diagnosis and extend after; or (2) start after diagnosis. Scenario 3: person-time will be considered as unexposed until the date of first prescription *following the date of cancer diagnosis* and as exposed thereafter. In all scenarios, cohort entry is date of index cancer diagnosis. All person-time of follow-up for patients who have never had a recorded prescription for a VGSC-inhibiting drug will be classified as unexposed.

The primary analysis will consider cancer-specific survival using adjusted Cox proportional hazards regression models. Right censoring will occur if the patient dies of any other cause, is still alive at the point the data were extracted or is transferred out of the CPRD practice. If we simply consider drug exposure in the model as a time-independent covariate where all person-time from cancer diagnosis to the end of follow-up (death or censor) is classified as ‘exposed’ for all patients with any VGSC-inhibiting drug prescriptions (whether started before or after diagnosis), immortal time bias^60^ will be introduced for patients whose prescriptions only start after their cancer diagnosis. To explore and account for this issue, drug exposure status will be considered as a time-dependent covariate in the following three ways (figure 1):

1. patients will be considered not to be exposed until the date they have their first prescription (whether before or after diagnosis) and will be assumed to be exposed thereafter. In this scenario, all person-time of follow-up from diagnosis to death/censor will be classified as exposed for patients who have a prescription *before* their cancer diagnosis (even those whose last prescription precedes diagnosis); while for those who only have prescriptions after their diagnosis, their survival time will be classified as unexposed between diagnosis and date of first prescription, and as exposed thereafter;
2. person-time will be considered as unexposed until the date of the first prescription and as exposed thereafter for patients whose prescriptions either: (1) start before diagnosis and extend after or (2) start after diagnosis;
3. a similar but stricter approach which more closely emulates the preclinical model^27^—person-time will be considered as unexposed until the date of the first prescription *following the date of cancer diagnosis* and as exposed thereafter.

In all scenarios, all person-time of follow-up for patients who have never had a recorded prescription for a VGSC-inhibiting drug will be classified as unexposed.

The distribution of time from diagnosis of cancer to death will be described using Kaplan-Meier survival estimates and Kaplan-Meier survival curves will be presented by drug exposure status. The statistical equivalence of the two curves will be tested using the log-rank test. Median time to death (or other appropriate quartile), with a 95% CI, will be presented.

### Sensitivity analysis

We will repeat the primary analyses adjusting the Cox models additionally for the following confounding variables: ethnicity, BMI, physical activity, smoking status, alcohol consumption, the Charlson Comorbidity Index and presence of a life-limiting indication for VGSC-inhibiting medication. In further sensitivity analyses, missing values for the confounding factors (previously included in a ‘not recorded’ category) will be imputed using multiple imputation within Stata where all the confounding variables and exposure to VGSC-inhibiting drugs are included in the models.

### Secondary analysis

We will consider survival stratified by cancer type using the same model specification as the primary analyses but omitting cancer type as a covariate (and gender among patients with prostate cancer).

We will repeat the primary analysis using as the ‘event’: (1) site-specific cancer listed among any of the causes of death; (2) any cancer listed among any of the causes of death and (3) all-cause mortality. Right censoring will occur if the patient dies of any other cause (except in case of all-cause mortality), is still alive at the end of follow-up or is transferred out of the CPRD practice.

### Type of drug and confounding indications

We shall consider the associations for each named VGSC-inhibiting drug by including patients in the exposed group for the drug that they were most commonly prescribed (where there are sufficient numbers of patients to do so).

The prescription data for participants with a recorded Read code for a life-limiting indication for a VGSC-inhibiting drug will be investigated, and patients with one of these diagnoses who do not have a recorded prescription for a VGSC-inhibiting drug will be identified and their alternative medications summarised. In order to investigate the mortality rates of patients with breast, bowel and prostate cancer with or without one of these conditions and taking VGSC-inhibiting drugs or not, the mortality of these four groups will be compared using death from any cancer and overall survival as the outcomes.

### Lidocaine injections

We will repeat the time-dependent analyses where the exposed group consists of patients whose VGSC-inhibiting drug prescriptions are only for lidocaine injections after diagnosis since there is evidence that local anaesthetics used perioperatively associates with reduced tumour recurrence.^55^

### Alternative cancers

In this study, as with our previous analysis, we shall initially focus on carcinomas of the breast, bowel and prostate as the role of VGSCs has been extensively studied in these types of cancer cells, and they are among the most common forms of cancer.^9^ ^10^ However, a benefit of this unrestricted data set is that we are able to investigate our hypotheses in patients diagnosed with other forms of cancer in which there is also evidence that VGSCs are expressed. We shall therefore repeat the primary analysis including patients with a recorded mention of lung cancer, melanoma, mesothelioma, glioma, cervical cancer, ovarian cancer, neuroblastoma and/or lymphoma.^11^
